# A nerve guidance conduit with topographical and biochemical cues: potential application using human neural stem cells

**DOI:** 10.1186/s11671-015-0972-6

**Published:** 2015-06-12

**Authors:** Phillip M Jenkins, Melissa R Laughter, David J Lee, Young M Lee, Curt R Freed, Daewon Park

**Affiliations:** Department of Bioengineering, University of Colorado Denver Anschutz Medical Campus, 12800 E. 19th Avenue, Aurora, CO 80045 USA; Division of Clinical Pharmacology and Toxicology, University of Colorado School of Medicine, 12700 E. 19th Avenue, Aurora, CO 80045 USA

**Keywords:** Biomimetic, Nerve regeneration, Electrospinning, Microchannel, Human neural stem cells

## Abstract

Despite major advances in the pathophysiological understanding of peripheral nerve damage, the treatment of nerve injuries still remains an unmet medical need. Nerve guidance conduits present a promising treatment option by providing a growth-permissive environment that 1) promotes neuronal cell survival and axon growth and 2) directs axonal extension. To this end, we designed an electrospun nerve guidance conduit using a blend of polyurea and poly-caprolactone with both biochemical and topographical cues. Biochemical cues were integrated into the conduit by functionalizing the polyurea with RGD to improve cell attachment. Topographical cues that resemble natural nerve tissue were incorporated by introducing intraluminal microchannels aligned with nanofibers. We determined that electrospinning the polymer solution across a two electrode system with dissolvable sucrose fibers produced a polymer conduit with the appropriate biomimetic properties. Human neural stem cells were cultured on the conduit to evaluate its ability to promote neuronal growth and axonal extension. The nerve guidance conduit was shown to enhance cell survival, migration, and guide neurite extension.

## Background

Functional recovery following a severe peripheral nerve injury is a daunting challenge in the field of neuroscience. After nerve transection, collapse and sealing of the axonal membranes at each nerve stump lead to the formation of either a growth cone or a retraction bulb. As part of the initial response after injury, myelin breaks down and axonal degeneration occurs at both the proximal and distal nerve stumps further increasing the gap between the nerve ends. Peripheral nerves are able to effectively regenerate over short distances; however, when the space between nerve ends is too wide (greater than 2 cm), surgical intervention is required to reconnect the damaged nerve using nerve grafts [1–3]. Currently, the gold standard for this type of peripheral nerve injury repair is surgical implantation of autologous nerve grafts. However, autographs have many clinical limitations including donor site morbidity, mismatch of donor size, neuropathic pain, and limited donor nerve length [4, 5].

To overcome these obstacles, considerable attention has been given to synthetic nerve conduits as an alternative to autographs. Synthetic nerve conduits are easily fabricated and show good reproducibility in structure specifications [5]. A variety of synthetic nerve conduits have been developed using different biomaterials such as polyamides [6], polyethylene terephthalate [7], poly(*L*-lactide) [8, 9], poly(ε-caprolactone) [10–12], poly(lactic glycolic acid) [13–15], and collagen [16, 17]. Blending of polymers has also been used to produce different types of conduits including simple hollow tubes [18], tubes with longitudinally aligned nanofibers along the interior lumen wall, and tubes with sheets of aligned nanofibers. Recently, more sophisticated conduits containing intraluminal microchannels of aligned nanofibers have shown promising results [19]. These aligned nanofibers and microchannels increase the surface area for which cells may interact with the conduit. However, these polymers rely solely on topographical cues to guide axonal growth and cell migration. It is likely that incorporating biochemical cues into these nerve conduits will further induce cell attachment and spreading, accelerating axonal extension during nerve regeneration [20–25].

For optimal nerve regeneration, synthetic conduits should include both topographical and biochemical cues. One commonly employed biochemical cue is Arg-Gly-Asp (RGD), a short peptide motif derived from the active sites of extracellular matrix (ECM) proteins specific for cell binding. RGD has been shown to enhance cell proliferation, migration, and survival in most tissues [26]. Previously, we reported that an RGD-functionalized, biomimetic poly(serinol hexamethylene urea) (PSHU) nanofiber conduit was shown to increase neurite guidance and axon extension in vitro [27]. In this study, the PSHU-RGD was further modified into a conduit with microchannels of aligned nanofibers. While the incorporation of RGD was intended to mimic biochemical cues of natural connective tissue found in nerves, the inclusion of microchannels with aligned nanofibers were intended to mimic the topographical cues in natural nerve tissue. Human neural stem cells (hNSCs) were used to evaluate the ability of this conduit to promote neurite guidance and extension. hNSCs are multipotent cells that primarily differentiate into neurons, oligodendrocytes, and astrocytes, making them ideal for modeling neural regeneration.

## Methods

### Equipment

All in vitro cell morphologies were examined on a Nikon DIAPHOT 300 equipped with CCD camera (SPOT RT 2.3.0, Diagnostic Instruments) using SPOT Advanced software for post hoc analysis and LSM 510 Laser Scanning Microscope. The microstructures of the electrospun nanofiber conduits were observed by field emission scanning electron microscopy (SEM) (JSM 7401F, JEOL).

### Materials

Trifluoroacetic acid (TFA), 2,2,2-trifluoroethanol (TFE), *N*-hydroxysuccinimide (NHS), *N*-(3-dimethylaminopropyl)-*N*′-ethylcarbodiimide hydrochloride (EDC), and 1,1,1,3,3,3-hexafluoro-2-propanol (HFP) were purchased from Alfa Aesar (Ward Hill, MA, USA). Anhydrous dichloromethane (DCM) was purchased from JT Baker (Phillipsburg, NJ, USA). The pentapeptide Gly-Arg-Gly-Asp-Ser (GRGDS) was purchased from Biomatik (Wilmington, DE, USA). *N,N*-dimethylformamide (DMF), hexamethylene diisocyanate (HDI), *N*-BOC-serinol, urea, and poly(ε-caprolactone) (PCL, M_n_: 70,000-90,000 g/mol) were purchased from Sigma-Aldrich (St. Louis, MO, USA). Anhydrous diethyl ether was purchased from Fisher Scientific (Pittsburgh, PA, USA).

### Synthesis of RGD conjugated PSHU (PSHU-RGD)

PSHU-RGD was synthesized as described previously [27]. Briefly, N-BOC-Serinol, HDI, and urea were reacted for 7 days at 90 °C. The PSHU was recovered by precipitation in diethyl ether. Following synthesis, the BOC groups were removed from PSHU by reaction with TFA/DCM (1:1) for 1 h at room temperature. Finally, RGD (1:1 molar ratio of RGD: −NH_2_ groups in PSHU) was conjugated to deprotected PSHU using EDC/NHS chemistry to yield PSHU-RGD.

### Scaffold synthesis by electrospinning

**Fig. 1:**
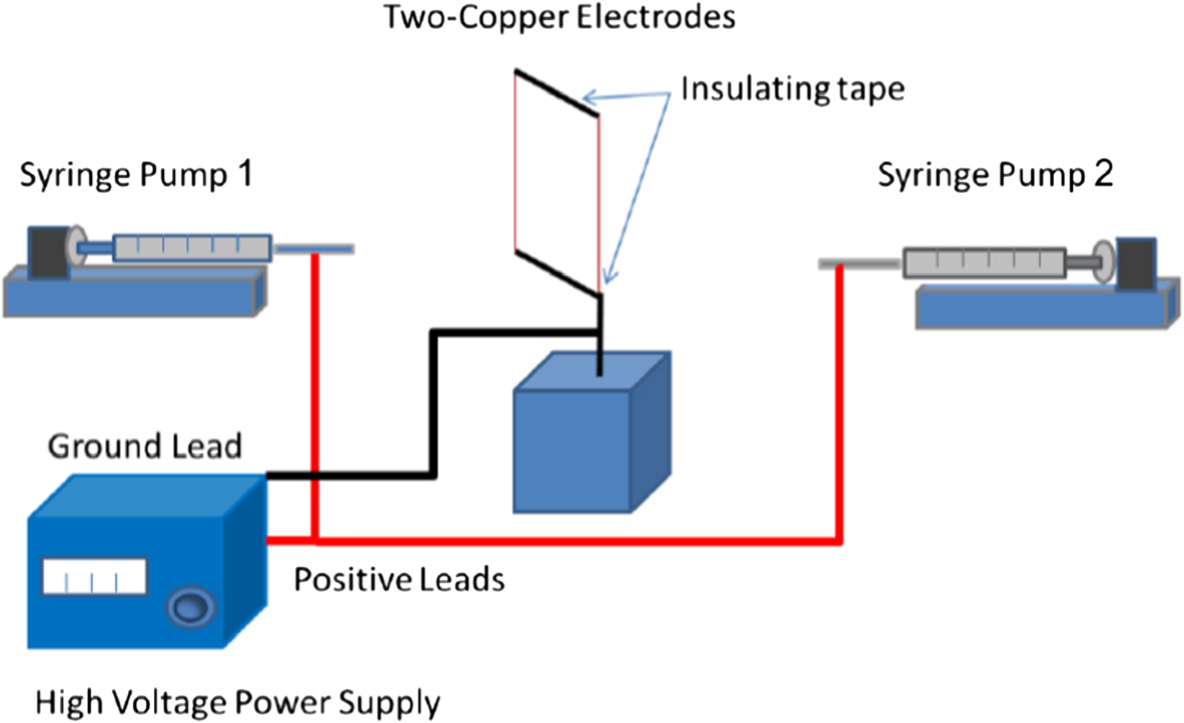
A schematic illustration of two-electrode electrospinning setup. An electric field perpendicular to the two electrodes is formed resulting in parallel nanofiber deposition across the gap. As more layers of aligned nanofibers are deposited across the gap, the electric charge distribution begins to shift, which leads to random nanofiber deposition on top of the layers of aligned nanofibers

Prior to electrospinning, sucrose fibers with diameters between 200 and 500 μm were formed using a fiber drawing method. Sucrose was heated to 75 °C until melted with a thick consistency. Then, the end of a microscope slide was dipped into the melted sucrose and fibers were drawn. The collector was constructed using two copper wire electrodes spaced 3.5 cm apart, and the sucrose fibers were fit to span the gap between the copper wire electrodes. Eight percent (*w*/*w*) polymer solutions in HFP were prepared for PSHU-RGD/PCL (30:70) blend, PSHU/PCL (30:70) blend, and pure PCL. The dual two-electrode electrospinning setup is depicted in Fig. 1. The collector was placed between the two needles distanced 10 cm from each needle, and the polymer solution was ejected at 1 ml/h through a 21-gauge stainless steel flat-tip needle at room temperature and relative humidity at 30 %. A positive 7.5-kV electrostatic potential was applied to both needles for the two blended solutions and a 9-kV potential for the pure PCL solution. For PSHU-RGD/PCL conduits, the PSHU-RGD/PCL blend was electrospun for the initial 15–20 min, and then the PSHU/PCL blend was used to deposit the remainder of the nanofibers. The pure PCL conduits were electrospun using its respective solution during the entire electrospinning process. The flat scaffold sheet was then allowed to air dry for 2 h before being removed from the collector, hand-rolled into a 1.2-mm-diameter tube, and cut into 1.5-cm-long conduits. Both ends of the conduits were loosely tied using nylon fishing line and then soaked in water for 48 h to dissolve out the sucrose fibers.

### Fiber angle distribution

OrientationJ, a plug-in for ImageJ, was used to process SEM images and generate a table of pixels counted versus degree of orientation. The data was then exported to Microsoft Office Excel 2010 and normalized to center the maximum peak at 0°. After normalization, the data was exported to Origin 9.1 to plot a histogram and to determine the degree of fiber angle agreement.

### Cell culture

All research met the requirements of the Helsinki Declaration. The method of induction of human neural stem cells from embryonic stem cells was modified from what was described previously [28]. Stem cell induction began with hESCs UCSF-4 cultured in mTesR media (STEMCELL) on Cellstart (Invitrogen) coating plates. UCSF-4 cells were passaged by cutting colonies into small squares using StemPro EZPassage Disposable Stem Cell Passaging Tool (Gibco) at a dilution of 1:10. For induction, UCSF-4 cells were treated with 10 ng/ml hLIF (Millipore), 3 μM CHIR99021 (Cellagentech), and 2 μM SB431542 (Cellagentech) in neural induction media, N2B27, containing DMEM/F12:neurobasal (1:1), 0.5× N2, 1× B27, 1 % Glutmax, for 10 days. The culture was then split 1:3 for the next passages using Accutase and expanded in neural induction media supplemented with 10 ng/ml hLIF, 3 μM CHIR99021, and 2 μM SB431542 on Cellstart coating plate.

### hNSC culture on nerve guidance channel

hNSCs (1 × 10^5^ cells/100 μl) were injected into the very tip of one end of 3 PSHU-RGD/PCL conduits and 3 PCL conduits and cultured on 24-well plates. After 24 h, 200 μM ascorbic acid and 10 ng/ml BDNF and GDNF were added to N2B27 culture media and changed daily. After a 14-day culture, conduits were washed twice in PBS then fixed at room temperature for 4 h with 4 % paraformaldehyde. Once fixed, the conduits were prepared for cryosectioning by soaking overnight at 4 °C in a 30 % sucrose PBS solution. After cryosectioning (20-μm slices), each section was washed twice in PBS for 10 min. Then, the samples were treated with blocking buffer (3 % goat serum PBS with 0.01 % Triton X-100) for 1 h at room temperature before adding β-III tubulin goat anti-mouse primary antibody (1:200 dilution, Promega, Madison, WI, USA) overnight at 4 °C. Following three PBS washes, the samples were incubated with anti-mouse secondary antibody conjugated to Alexa 488 (1:300 dilutions, Invitrogen) for 1 h at room temperature in the dark. The samples were examined using confocal microscopy.

## Results and discussion

The effect of RGD as a biochemical cue has been previously examined with PC12 cells and hNSCs. PC12 cells on surfaces coated with PSHU-RGD showed significantly higher levels of cell attachment, differentiation, and neurite outgrowth compared to surfaces coated without the presence of RGD [27]. An increase in hNSC survival and differentiation was observed on PSHU-RGD surfaces compared to surfaces without RGD [29]. In this study, a biochemically and topographically controlled synthetic nerve conduit was developed with emphasis on promoting neuronal cell survival, migration, and guided extension. After confirmation of the conduit structure and the presence of aligned microchannels, the conduits were assessed in vitro by hNSC cultures.

### Scaffold synthesis by electrospinning

Dual two-electrode electrospinning has been shown to produce highly aligned nanofibers but utilizes a double nozzle system to produce a composite material. Our initial design was to fabricate a conduit with PSHU-RGD alone; however, PSHU-RGD nanofibers proved too brittle to be further processed into the desired conduit. Thus, to provide more flexibility, PSHU-RGD was blended with PCL.

**Fig. 2:**
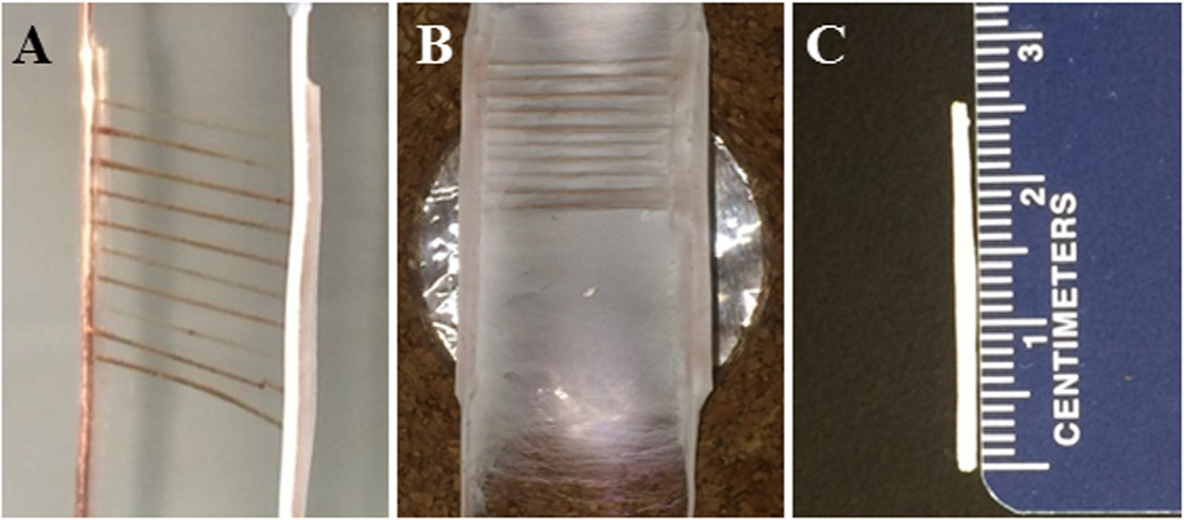
Nerve guidance conduit fabricated by the two-electrode electrospinning setup: **a** the two-electrode template with perpendicularly bridged sucrose fibers, **b** after electrospinning the nanofiber scaffold was allowed to air dry for 2 h, and **c** the scaffold was cut away from the electrodes and rolled into a tubular conduit

Initially, PSHU-RGD/PCL was electrospun to the two-electrode template with sucrose fibers perpendicularly bridged to the electrodes (Fig. 2a). A 30:70 (PSHU-RGD/PCL) ratio was determined to produce a solution with favorable viscosity for electrospinning and yielded the desired nanofiber configurations. At the start of electrospinning, the nanofiber deposition was aligned parallel to the sucrose fibers. After observing 15–20 min of aligned nanofiber deposition, random nanofiber deposition began to form on top of the aligned nanofibers, a phenomenon which actually increases the structural strength and integrity of the microchannels and final conduit. At this point, the polymer solution was switched from PSHU-RGD/PCL to PSHU/PCL so that biochemical cues would not be present on the remainder of the random nanofibers (Fig. 2b). Finally, the scaffold was successfully rolled up to form a tubular conduit (Fig. 2c).

**Fig. 3:**
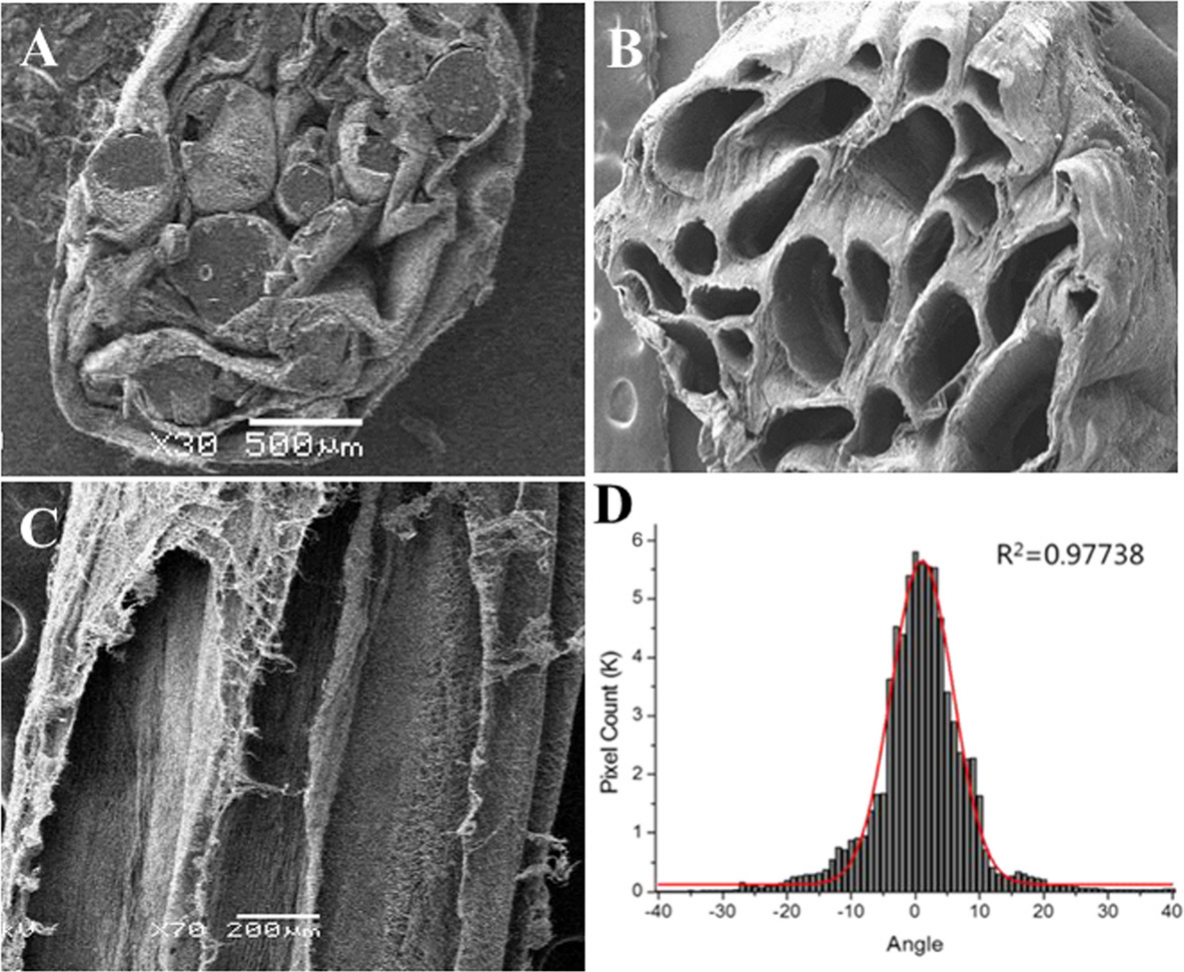
SEM images of PSHU-RGD/PCL conduit: **a** cross section with the embedded sucrose fibers, **b** cross section with microchannels after dissolving out sucrose fibers, **c** longitudinal section showing highly aligned nanofibers inside the microchannels, and **d** fiber angle distribution inside the microchannels normalized to 0°

SEM image analysis of the conduit’s cross section showed that the sucrose fibers were successfully embedded within the conduit (Fig. 3a). The sucrose was then dissolved out of the conduit to expose the microchannels (Fig. 3b,c). Importantly, these properties resembled the natural nerve structure of the epineurium and perineurium and therefore may mimic the topographical cues of natural nerve tissues. In addition, the lumen of the microchannels also presented highly aligned structures with uniform orientation within ±5° angles when normalized to 0° (Fig. 3d), which was expected to play a pivotal role in guided axonal growth.

Neurite extension and fiber alignment has been previously examined using PC12 cells. PC12 cells seeded on randomly aligned nanofibers had highly branched and randomly aligned neurite outgrowth. In contrast, PC12 cells seeded on aligned fibers were less branched, sprouted along the direction of the aligned fibers, and extended further from the cell bodies, similarly to native neurons [27].

### hNSC response to nerve guidance conduit

Stem cell research has gained significant interest in the field of nerve regeneration. However, one obstacle to these cell-based therapies is the lack of a sufficient cell scaffold or conduit to enhance the survival and axon extension of the implanted cells [30]. Through the presence of both topographical and biochemical cues, we expect that our conduit may overcome these obstacles and serve as a nerve guide for cell-based therapies. hNSCs were cultured on the nerve guidance conduits to determine if cell migration and neurite extension are enhanced.

**Fig. 4:**
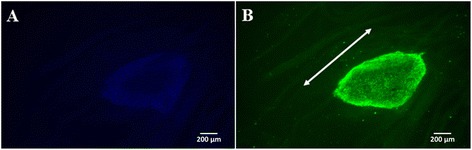
Fluorescence microscopy images of hNSCs cultured with PCL conduit for 14 days: **a** DAPI and **b** β-III tubulin. DAPI staining is indicated in blue and β-III tubulin staining is indicated in *green. Arrow* indicates the direction of aligned nanofibers

We began by testing the ability of the PCL conduit to encourage cell growth and axon extension. Fluorescent microscopy was initially used to observe cell attachment. Interestingly, sparsely distributed and aggregated hNSCs were found in PCL conduit with no directional preference (Fig. 4). We speculate that although the pure PCL conduit provided topographical cues through the presence of aligned microchannels, the hNSCs did not extend axons through the conduit due to the absence of biochemical cues. Since hNSC survival, migration, and guided extension with the PCL conduit was not observed, no further examination by confocal microscopy was performed.

**Fig. 5:**
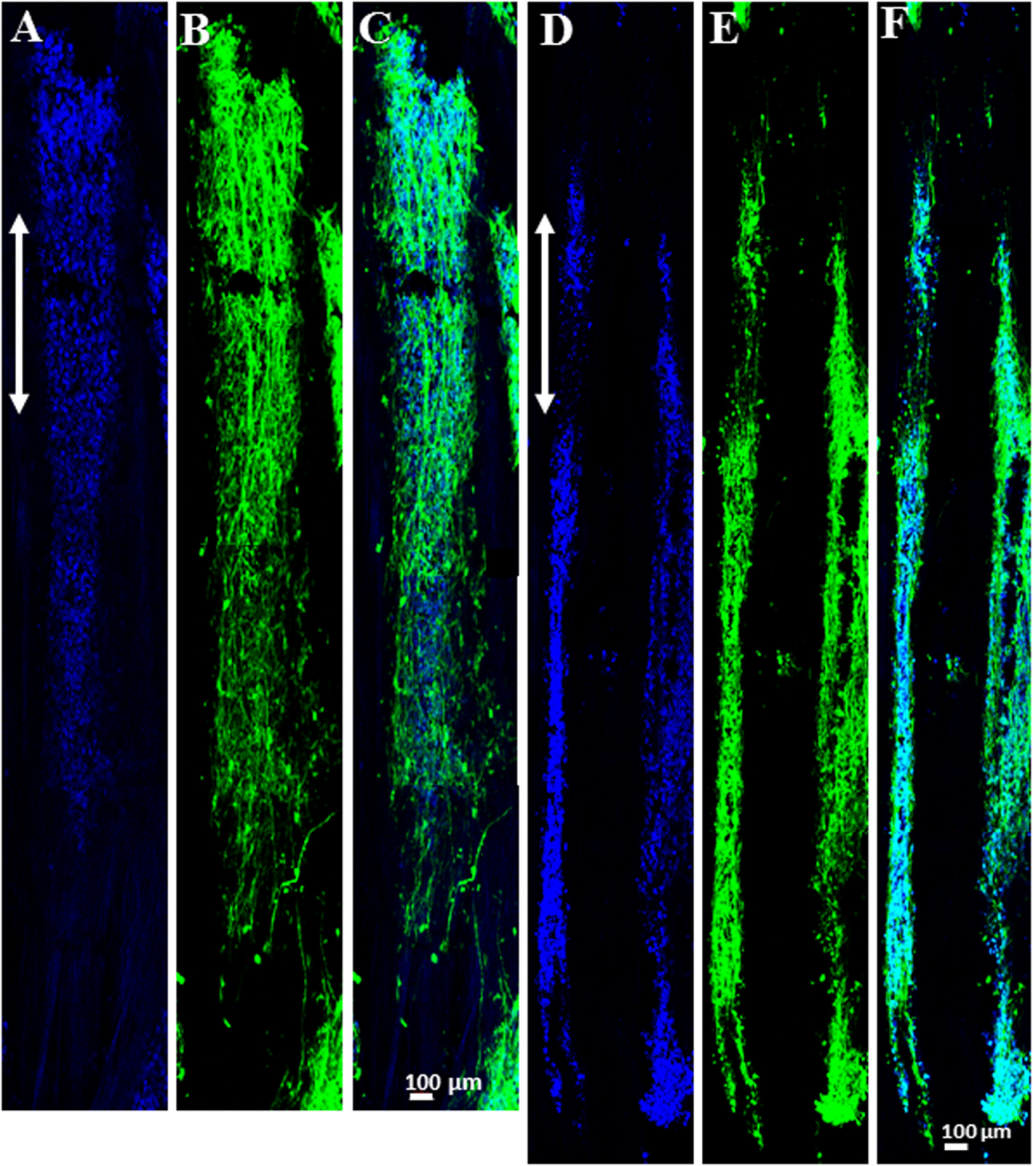
Confocal microscopy images of hNSCs cultured with PSHU-RGD/PCL conduit for 14 days: representative images **a**, **b** along the center and **e**, **f** inner wall of microchannels, **a**, **d** DAPI, **b**, **e** β-III tubulin, and **c**, **f** combined DAPI and β-III tubulin images. DAPI staining is indicated in *blue* and β-III tubulin staining is indicated in *green*. Images show that hNSCs migrated far into the conduit with extensive neurite extension along the nanofiber direction (*arrows*)

Next, we tested the ability of the PSHU-RGD/PCL conduit to induce hNSC growth and neurite extension. Florescent microscopy was used to confirm cell attachment, and confocal microscopy was used for further investigation. In contrast, a significantly higher cell density was found in the PSHU-RGD/PCL conduit after the culture period (Fig. 5). In addition, the cells migrated into the microchannels (2 mm) with considerable neurite extension. hNSC migration across the lumen of the grafts is apparent as the cells were introduced at the very tip of one end of the graft and hNSC activity is observed across the length of the conduit. Representative section images were taken along the center (Fig. 5a–c) and the inner wall (Fig. 5d–f) of microchannels. We observed the same hNSC behavior in all microchannels. In addition, hNSC survival, migration, and guided neurite extension were observed with the PSHU-RGD/PCL conduit. These findings provide evidence that the PSHU-RGD/PCL conduit offers a microenvironment conducive to hNSC survival, migration, and extension.

## Conclusions

A nerve guidance conduit was successfully fabricated for applications in neuronal tissue engineering. This conduit was designed to provide 1) biochemical cues through the conjugation of RGD and 2) topographical cues through microchannels with aligned nanofibers. The topographical and biochemical cues present on the PSHU-RGD/PCL conduit were shown to encourage hNSC growth and neurite extension when compared to the pure PCL conduit. These findings could have a significant impact on current nerve grafts and the treatment of peripheral nerve damage. Furthermore, the design concept of this conduit could provide a platform for improved nerve regeneration with enhanced cell survival, migration, and guided extension.

## References

[1] Bradke F, Fawcett JW, Spira ME (2012). Assembly of a new growth cone after axotomy: the precursor to axon regeneration. Nat Rev Neurosci..

[2] Deumens R, Bozkurt A, Meek MF, Marcus MAE, Joosten EAJ, Weis J, et al. Repairing injured peripheral nerves: bridging the gap. Prog Neurobiol [Internet]. Elsevier Ltd; 2010;92(3):245–76. Available from: http://dx.doi.org/10.1016/j.pneurobio.2010.10.002. 10.1016/j.pneurobio.2010.10.002 20950667

[3] Navarro X, Vivó M, Valero-Cabré A (2007). Neural plasticity after peripheral nerve injury and regeneration. Progr Neurobiol..

[4] Panseri S, Cunha C, Lowery J, Del Carro U, Taraballi F, Amadio S (2008). Electrospun micro- and nanofiber tubes for functional nervous regeneration in sciatic nerve transections. BMC Biotechnol..

[5] Yu W, Zhao W, Zhu C, Zhang X, Ye D, Zhang W, et al. Sciatic nerve regeneration in rats by a promising electrospun collagen/poly(ε-caprolactone) nerve conduit with tailored degradation rate. BMC Neurosci [Internet]. BioMed Central Ltd; 2011;12(1):68. Available from: http://www.biomedcentral.com/1471-2202/12/68. 10.1186/1471-2202-12-68 PMC3148572 21756368

[6] Yannas IV, Hill BJ (2004). Selection of biomaterials for peripheral nerve regeneration using data from the nerve chamber model. Biomaterials..

[7] Zang R, Yang S-T. Multiwalled carbon nanotube-coated polyethylene terephthalate fibrous matrices for enhanced neuronal differentiation of mouse embryonic stem cells. J Mater Chem B [Internet]. The Royal Society of Chemistry; 2013 Jan 3 [cited 2015 Feb 24];1(5):646. Available from: http://pubs.rsc.org/en/content/articlehtml/2013/tb/c2tb00157h. 10.1039/c2tb00157h 32260768

[8] Hsu SH, Chan SH, Chiang CM, Chi-Chang Chen C, Jiang CF (2011). Peripheral nerve regeneration using a microporous polylactic acid asymmetric conduit in a rabbit long-gap sciatic nerve transection model. Biomaterials..

[9] Quigley a F, Bulluss KJ, Kyratzis ILB, Gilmore K, Mysore T, Schirmer KSU, et al. Engineering a multimodal nerve conduit for repair of injured peripheral nerve. J Neural Eng [Internet]. 2013;10:016008. Available from: http://www.ncbi.nlm.nih.gov/pubmed/23283383. 10.1088/1741-2560/10/1/016008 23283383

[10] Jha BS, Colello RJ, Bowman JR, Sell SA, Lee KD, Bigbee JW (2011). Two pole air gap electrospinning: fabrication of highly aligned, three-dimensional scaffolds for nerve reconstruction. Acta Biomater..

[11] Jiang X, Mi R, Hoke A, Chew SY (2014). Nanofibrous nerve conduit-enhanced peripheral nerve regeneration. J Tissue Eng Regen Med..

[12] Reid AJ, de Luca AC, Faroni A, Downes S, Sun M, Terenghi G (2013). Long term peripheral nerve regeneration using a novel PCL nerve conduit. Neurosci Lett..

[13] Chang CJ, Hsu SH, Yen HJ, Chang H, Hsu SK (2007). Effects of unidirectional permeability in asymmetric poly(DL-lactic acid-co-glycolic acid) conduits on peripheral nerve regeneration: an in vitro and in vivo study. J Biomed Mater Res - Part B Appl Biomater..

[14] Fan J, Zhang H, He J, Xiao Z, Chen B, Xiaodan J (2011). Neural regrowth induced by PLGA nerve conduits and neurotrophin-3 in rats with complete spinal cord transection. J Biomed Mater Res - Part B Appl Biomater.

[15] Sasaki R, Aoki S, Yamato M, Uchiyama H, Wada K, Ogiuchi H (2011). PLGA artificial nerve conduits with dental pulp cells promote facial nerve regeneration. J Tissue Eng Regen Med..

[16] Berrocal Y a, Almeida VW, Gupta R, Levi AD. Transplantation of Schwann cells in a collagen tube for the repair of large, segmental peripheral nerve defects in rats. J Neurosurg [Internet]. 2013;119:720–32. Available from: http://www.ncbi.nlm.nih.gov/pubmed/23746104. 10.3171/2013.4.JNS121189 23746104

[17] Pertici V, Laurin J, Féron F, Marqueste T, Decherchi P (2014). Functional recovery after repair of peroneal nerve gap using different collagen conduits. Acta Neurochir (Wien)..

[18] Liu B, Cai SX, Ma KW, Xu ZL, Dai XZ, Yang L (2008). Fabrication of a PLGA-collagen peripheral nerve scaffold and investigation of its sustained release property in vitro. J Mater Sci Mater Med..

[19] Jeffries EM, Wang Y. Incorporation of parallel electrospun fibers for improved topographical guidance in 3D nerve guides. Biofabrication [Internet]. 2013;5:035015. Available from: http://www.ncbi.nlm.nih.gov/pubmed/23945055. 10.1088/1758-5082/5/3/035015 23945055

[20] Griffith LG, Naughton G (2002). Tissue engineering–current challenges and expanding opportunities. Science..

[21] Hench LL, Polak JM (2002). Third-generation biomedical materials. Science..

[22] Langer R, Tirrell DA (2004). Designing materials for biology and medicine. Nature..

[23] Sakiyama-Elbert SE, Hubbell JA (2001). Functional biomaterials: design of novel biomaterials. Annu Rev Mater Res [Internet]..

[24] Shin H, Jo S, Mikos AG. Biomimetic materials for tissue engineering. Biomaterials [Internet]. 2003 Nov [cited 2014 Jul 10];24(24):4353–64. Available from: http://www.sciencedirect.com/science/article/pii/S0142961203003399. 10.1016/s0142-9612(03)00339-9 12922148

[25] Wang M, Zhai P, Chen X, Schreyer DJ, Sun X, Cui F (2011). Bioengineered scaffolds for spinal cord repair. Tissue Eng Part B Rev..

[26] Zhang P, Wu H, Wu H, Lù Z, Deng C, Hong Z (2011). RGD-conjugated copolymer incorporated into composite of poly(lactide-co-glycotide) and poly(l-lactide)-grafted nanohydroxyapatite for bone tissue engineering. Biomacromolecules..

[27] Yun D, Famili A, Lee YM, Jenkins PM, Freed CR, Park D. Biomimetic poly(serinol hexamethylene urea) for promotion of neurite outgrowth and guidance. J Biomater Sci Polym Ed [Internet]. 2014;25(March):354–69. Available from: http://www.ncbi.nlm.nih.gov/pubmed/24279744. 10.1080/09205063.2013.861170 24279744

[28] Li W, Sun W, Zhang Y, Wei W, Ambasudhan R, Xia P (2011). Rapid induction and long-term self-renewal of primitive neural precursors from human embryonic stem cells by small molecule inhibitors. Proc Natl Acad Sci U S A..

[29] Yun D, Lee YM, Laughter MR, Freed CR, Park D. Substantial differentiation of human neural stem cells into motor neurons on a biomimetic polyurea. Macromol Biosci. 2015;15. doi:10.1002/mabi.201500066. 10.1002/mabi.201500066 PMC4558270 26033933

[30] Lavasani M, Thompson SD, Pollett JB, Usas A, Lu A, Stolz DB (2014). Human muscle-derived stem/progenitor cells promote functional murine peripheral nerve regeneration. J Clin Invest..

